# Association of Uncontrolled Hypertension or Diabetes Mellitus With Major Adverse Cardiovascular Events and Mortality in South Korea: Population-Based Cohort Study

**DOI:** 10.2196/42190

**Published:** 2023-02-03

**Authors:** Sung-Hee Oh, Dohyang Kim, Jinseub Hwang, Jae-Heon Kang, Yeongkeun Kwon, Jin-Won Kwon

**Affiliations:** 1 BK21 FOUR Community-Based Intelligent Novel Drug Discovery Education Unit College of Pharmacy and Research Institute of Pharmaceutical Sciences Kyungpook National University Daegu Republic of Korea; 2 Department of Statistics, Daegu University Gyeongbuk Republic of Korea; 3 Department of Family Medicine, Kangbuk Samsung Hospital, College of Medicine, Sungkyunkwan University Seoul Republic of Korea; 4 Centre for Obesity and Metabolic Diseases, Korea University Anam Hospital Seoul Republic of Korea

**Keywords:** prevention and control, cardiovascular diseases, diabetes mellitus, hypertension, extended Cox regression, cohort study

## Abstract

**Background:**

Managing hypertension (HT) and diabetes mellitus (DM) is crucial to preventing cardiovascular diseases. Few studies have investigated the incidence and risk of cardiovascular diseases or mortality in uncontrolled HT or DM in the Asian population. Epidemiological studies of cardiovascular disease should be conducted with continuous consideration of the changing disease risk profiles, lifestyles, and socioeconomic status over time.

**Objective:**

We aimed to examine the association of uncontrolled HT or DM with the incidence of cardiovascular events or deaths from any cause.

**Methods:**

This population-based retrospective study was conducted using data from the Korean National Health Insurance Service–National Health Screening Cohort, including patients aged 40-79 years who participated in national screening from 2002 to 2003 and were followed up until 2015. The health screening period from 2002 to 2013 was stratified into 6 index periods in 2-year cycles, and the follow-up period from 2004 to 2015 was stratified accordingly into 6 subsequent 2-year periods. The incidence rates and hazard ratio (HR) for major adverse cardiovascular events (MACE) and death from any cause were estimated according to HT or DM control status. Extended Cox models with time-dependent variables updated every 2 years, including sociodemographic characteristics, blood pressure (BP), fasting blood glucose (FBG), medication prescription, and adherence, were used.

**Results:**

Among the total cohort of 440,249 patients, 155,765 (35.38%) were in the uncontrolled HT or DM group. More than 60% of the patients with HT or DM who were prescribed medications did not achieve the target BP or FBG. The incidence of MACE was 10.8-15.5 and 9.6-13.3 per 1000 person-years in the uncontrolled DM and uncontrolled HT groups, respectively, and increased with age. In the uncontrolled HT and DM group, the incidence of MACE was high (15.2-17.5 per 1000 person-years) at a relatively young age and showed no age-related trend. Adjusted HR for MACE were 1.28 (95% CI 1.23-1.32) for the uncontrolled DM group, 1.32 (95% CI 1.29-1.35) for the uncontrolled HT group, and 1.54 (95% CI 1.47-1.60) for the uncontrolled HT and DM group. Adjusted HR for death from any cause were 1.05 (95% CI 1.01-1.10) for the uncontrolled DM group, 1.13 (95% CI 1.10-1.16) for the uncontrolled HT group, and 1.17 (95% CI 1.12-1.23) for the uncontrolled HT and DM group.

**Conclusions:**

This up-to-date evidence of cardiovascular epidemiology in South Korea serves as the basis for planning public health policies to prevent cardiovascular diseases. The high uncontrolled rates of HT or DM, regardless of medication prescription, have led us to suggest the need for a novel system for effective BP or glycemic control, such as a community-wide management program using mobile health technology.

## Introduction

Cardiovascular diseases impose enormous health and economic burden globally [1]. The World Health Organization reported that approximately 17.9 million people died of cardiovascular diseases in 2019, accounting for 32% of deaths worldwide [2]. In South Korea, 17.8% of the total deaths reported in 2019 were caused by heart diseases (10.5%) and cerebrovascular diseases (7.3%) [3]. The substantial burden of cardiovascular diseases in the recent years is attributable to the increase in the number of patients living with cardiovascular diseases, prolonged duration of diseases, and accompanying disabilities [4,5]. The global burden of cardiovascular diseases, evaluated as disability-adjusted life years, has increased by 46% for ischemic heart disease (IHD) and by 32% for stroke from 1990 to 2019, with IHD ranking first and stroke ranking second in the global burden of disease among adults aged 50 to 74 years in 2019 [6].

To prevent cardiovascular diseases, it is important to manage hypertension (HT) and diabetes mellitus (DM), known as the most common contributing risk factors [7]. However, approximately half of the adults with high blood pressure (BP) or blood glucose level remain undiagnosed [8,9]. Furthermore, the target BP and blood glucose levels may not be achieved even with treatment owing to nonadherence to therapy or unhealthy lifestyles [10]. The uncontrolled rate among people with HT in 2019 was reported to be approximately 80% globally, and approximately 50% of the patients undergoing treatment did not achieve BP control [8]. In addition, a study in the United States found that most patients with diabetes were treated but the uncontrolled rate was approximately 50% [11].

A few studies have reported the incidence and risk of complications according to the control status of patients diagnosed with HT or DM. In a recent study, patients with uncontrolled DM had 68% higher odds of developing micro- or macroangiopathy than those with controlled DM [12]. Furthermore, individuals with undiagnosed DM have poor long-term glycemic control and can die within a short time after the onset of complication-related symptoms [13]. The incidence of cardiovascular and cerebrovascular complications was higher in patients with refractory HT on treatment than in those with controlled HT [14]. The data on recent population-based epidemiological studies conducted in the Asian population are relatively scarce [15]. Uncontrolled HT or DM may increase the incidence of cardiovascular events or death regardless of the medication prescription or diagnosis of HT or DM.

The incidence, mortality, and risk of cardiovascular disease vary substantially between populations and over time [16], and it is important to investigate the epidemiology of cardiovascular disease by continuously considering the changing disease risk profiles, lifestyles, and socioeconomic status over time [6]. Therefore, the aim of this nationwide study was to determine the incidence of cardiovascular events according to HT or DM control status in South Korea and to examine the association between disease control and cardiovascular events or death from any cause by adjusting for time-varying characteristics.

## Methods

### Data Sources and Study Population

This retrospective population-based cohort study was based on data from the National Health Insurance Service–National Health Screening Cohort (NHIS-HEALS) in South Korea. The NHIS-HEALS sample data included 514,866 individuals (aged 40-79 years), who comprised a random selection of 10% of all national health screening program participants covered under the mandatory National Health Insurance or Medical Aid system in 2002 and 2003. The cohort was followed up to 2015 annually for information on death and health care use and biennially for the health screening information. The NHIS-HEALS data provided the following information: demographic variables, income, and date of death from the eligibility database; variables for specific health problems and risk factors evaluated using a self-administered questionnaire such as smoking status, frequency per week of alcohol consumption, days per week of physical activity, medical history and family history, and bioclinical laboratory results from the national health screening database; records of inpatient and outpatient use, including procedures, prescribed drugs, treatments, and diagnostic information using the International Classification of Diseases, 10th revision code from the health care use database based on data collected during the process of claiming health care services; and the types of health care institutions and health care human resources from the health care provider database. These 4 databases were linked using unique personal identification numbers created for the NHIS-HEALS [17]. Among the 514,866 individuals, 489,276 (95.03%) with at least 1 claim of health care services use between 2002 and 2003 were included in the study to define the baseline clinical information. Study participants with cardiovascular disease or all-cause death from 2002 to 2003 were excluded (n=48,217), and 810 participants with missing data on BP and fasting blood glucose (FBG) from 2002 to 2003 were excluded. After exclusion, a total of 440,249 participants were included in the study (Figure S1 in Multimedia Appendix 1).

### Ethical Consideration

This study was exempted from the Institutional Review Board of Kyungpook National University (number 2020-0157) because the data used in this study were anonymized and deidentified. Informed consent was not required owing to the retrospective nature of the study.

### Study Design and Measurements

**Figure 1:**
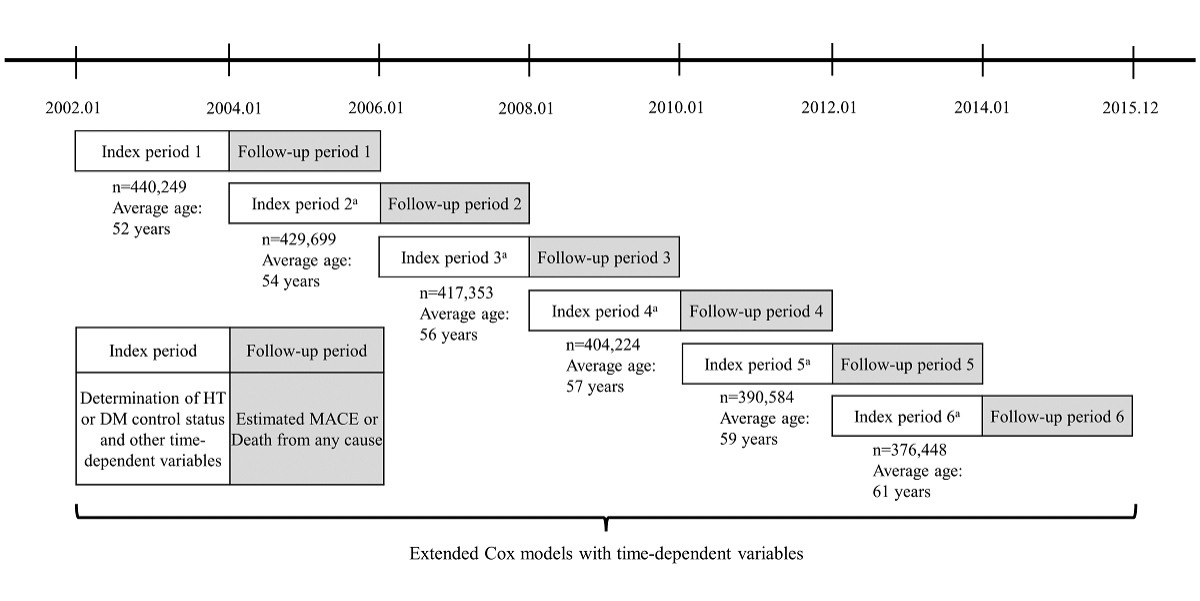
Schematic description of the study design. The cohort of national health screening program participants from 2002 to 2003 was followed up to 2015. The health screening period of a 2-year cycle from 2002 to 2013 was defined as 6 index periods, and the follow-up period from 2004 to 2015 was stratified according to 6 subsequent 2-year periods after each index period. ^a^Participants with MACE or death from any cause during the previous follow-up period were excluded from the participants for the subsequent index period. From index periods 1 to 6, the number of participants and the average age were presented. DM: diabetes mellitus; HT: hypertension; MACE: major adverse cardiovascular events.

The health screening period from 2002 to 2013 was stratified into 6 index periods in 2-year cycles, and the bioclinical laboratory results and various confounding variables were determined during the index period. The follow-up period from 2004 to 2015 was stratified accordingly into six 2-year periods. The subsequent 2-year periods after each index period were defined as follow-up periods 1 to 6. Cardiovascular disease and death from any cause were estimated for each group based on the control status of HT or DM during the 6 follow-up periods (Figure 1). The bioclinical laboratory tests (BP, FBG, BMI, etc) were conducted on participants after an overnight fast at the biennial health screening examination. BP was measured in the seated participants for approximately 5 minutes, and HT was defined as systolic BP ≥140 mm Hg and diastolic BP ≥90 mm Hg. DM was defined as an FBG level ≥126 mg/dL. Well-controlled status was defined as FBG level <126 mg/dL for DM and systolic BP <140 mm Hg and diastolic BP <90 mm Hg for HT. As confounding variables associated with an increased risk of cardiovascular diseases, this study considered age; sex; type of national health security program; income level; BMI; smoking; drinking; exercise; Charlson Comorbidity Index (CCI); disability severity; number of outpatient visits and hospitalization days; use of medication; and medication possession ratio (MPR) of antihypertensive, antidiabetic, and antihyperlipidemic medications [4]. All drugs listed in the national formulary in South Korea as antidiabetic, antihypertensive, and antihyperlipidemic medications were included. Medication users were defined as those who were prescribed medication at least once. Age was classified as middle age (40-64 years) and older age (65-69 years, 70-79 years, and ≥80 years). The income levels were divided into grades 0 to 2 (lowest), 3 to 5, 6 to 8, and 9 to 10 (highest). A person with a grade of 0 covered under the medical aid system was defined as having an income below the minimum cost of living. Study participants were categorized according to BMI based on the World Health Organization criteria for the Asian population: <18.5 kg/m^2^ (underweight), 18.5 to 24.9 kg/m^2^ (normal to overweight), 25.0 to 29.9 kg/m^2^ (class 1 obese), or ≥30 kg/m^2^ (class 2 obese) [18]. Disability severity was defined as severe (grades 1 to 3), mild (grades 4 to 6), and no disability, based on the disability grade system under the Welfare of Disabled Persons Act. MPR was defined as the sum of the days of supply from the first to the last prescription divided by the time between the last prescription date plus the days of supply and the first prescription date [19].

### Outcomes

Study outcomes included (1) any major adverse cardiovascular events (MACE), that is, hospitalization for cardiovascular disease defined as a composite of IHD (myocardial infarction and angina), cerebrovascular diseases (cerebral infarction, hemorrhagic stroke, and transient ischemic attack), and heart failure; (2) death from any cause; and (3) MACE or death from any cause [20]. We defined MACE using the primary and secondary International Classification of Diseases, 10th revision diagnostic codes listed in Table S1 in Multimedia Appendix 1. All study participants underwent follow-up examinations until the occurrence of either of the following 2 outcomes: incidence of MACE or death from any cause or the end of follow-up in 2015, whichever occurred first.

### Statistical Analysis

The characteristics of the 2 groups were compared using the chi-square test for categorical variables, indicated as count with percentage, and 2-tailed *t* test for continuous variables, indicated as mean with SD.

Incidence rates over each period were calculated as the number of events per 1000 person-years of observation for each outcome; the rates were standardized to the age distribution from each period. Numerators were the number of first events in a specified follow-up period, and denominators were the number of persons at risk during the corresponding index period. Data on individuals who died were censored at the time of death. Extended Cox models with time-dependent variables were used to estimate the hazard ratio (HR) for the association of HT or DM control with MACE or death from any cause. The estimated risk factor measurements, such as BP, FBG, age, type of national health security program, income level, BMI, smoking, drinking, exercise, CCI, disability severity, number of outpatient visits and hospitalization days, and MPR, were used as time-dependent variables. These variables were updated every 2 years. If participants had a missing value in an index period, it was replaced by that participant’s previously observed value using the last observation carried forward imputation method. Statistical analyses were conducted using SAS software (version 9.4; SAS Institute Inc) at a significance level of .05.

## Results

### Characteristics

Among the total cohort of 440,249 individuals, 284,484 (64.61%) individuals were in the well-controlled group and 155,765 (35.38%) individuals were in the uncontrolled group. The average age at entry was 52 (SD 9.4) years for all participants, which increased from 52 to 61 years during index periods 1 to 6 (Figure 1). The characteristics of the study population during the first index period are presented in Table 1. There were substantial differences between the well-controlled and uncontrolled groups in age, sex, income level, national health security program, BMI, smoking, drinking frequency, exercise frequency, CCI, disability severity, number of outpatient visits and hospitalization days, and medication use (*P*<.001). The uncontrolled group had a higher proportion of men, obesity, current smokers, and patients with CCI ≥3; higher drinking frequency; and lower income levels than those in the well-controlled group. Only 23.24% (36,192/155,765) of all uncontrolled patients were prescribed medication; the proportion of patients with antihypertensive medication use was 18.89% (23,169/122,646) in the uncontrolled HT group, and the proportion of patients with antidiabetic medication use was 28.41% (5126/18,042) in the uncontrolled DM group. Among the uncontrolled patients, despite being prescribed medications, 34.92% (10,380/29,523) of patients who had been prescribed antihypertensive medications and 39.12% (4784/12,229) of patients who had been prescribed antidiabetic medications had an MPR <70%. Meanwhile, >60% of medication users had uncontrolled HT or DM: 64.05% (27,318/42,652) of patients who had been prescribed antihypertensive medications had uncontrolled HT and 60.85% (9388/15,429) of patients who had been prescribed antidiabetic medications had uncontrolled DM.

**Table 1:** Characteristics by the control status of hypertension (HT) or diabetes mellitus (DM) of study participants in the first index period in 2002 to 2003.

Characteristics				Well-controlled group (n=284,484)		Uncontrolled group						
						HT (n=122,646)		DM (n=18,042)		HT and DM (n=15,077)		Total (n=155,765)^a^
**Age (years), n (%)**												
	40 to <65			257,700 (90.59)		99,589 (81.2)		14,920 (82.70)		11,642 (77.22)		126,151 (80.99)
	65 to <70			16,147 (5.68)		12,985 (10.59)		1817 (10.07)		1877 (12.45)		16,679 (10.71)
	70 to <80			10,637 (3.74)		10,072 (8.21)		1305 (7.23)		1558 (10.33)		12,935 (8.3)
**Sex, n (%)**												
	Male			142,312 (50.02)		71,289 (58.13)		11,246 (62.33)		9793 (64.95)		92,328 (59.27)
	Female			142,172 (49.98)		51,357 (41.87)		6796 (37.67)		5284 (35.05)		63,437 (40.73)
**Income level, n (%)**												
	Lowest			42,429 (14.91)		21,226 (17.31)		3034 (16.82)		2823 (18.72)		27,083 (17.39)
	Low-middle			60,505 (21.27)		28,554 (23.28)		4129 (22.89)		3571 (23.69)		36,254 (23.27)
	Middle-high			81,798 (28.75)		35,662 (29.08)		5318 (29.48)		4469 (29.64)		45,449 (29.18)
	Highest			99,752 (35.06)		37,204 (30.33)		5561 (30.82)		4214 (27.95)		46,979 (30.16)
**National health security program, n (%)**												
	National Health Insurance			284,284 (99.93)		122,517 (99.89)		18,012 (99.83)		15,062 (99.9)		155,591 (99.89)
	Medical Aid			200 (0.07)		129 (0.11)		30 (0.17)		15 (0.1)		174 (0.11)
**BMI** **(kg/m^2^), n (%)**												
	<18.5			7657 (2.69)		2073 (1.69)		401 (2.22)		214 (1.42)		2688 (1.73)
	≥18.5 to <25			193,630 (68.06)		66,960 (54.6)		10,724 (59.44)		7447 (49.39)		85,131 (54.65)
	≥25 to <30			78,138 (27.47)		48,352 (39.42)		6319 (35.02)		6507 (43.16)		61,178 (39.28)
	≥30			5059 (1.78)		5261 (4.29)		598 (3.31)		909 (6.03)		6768 (4.35)
**Smoking, n (%)**												
	Nonsmoker			188,771 (66.36)		78,327 (63.86)		10,489 (58.14)		8952 (59.38)		97,768 (62.77)
	Ex-smoker			22,882 (8.04)		11,157 (9.1)		1607 (8.91)		1367 (9.07)		14,131 (9.07)
	Current smoker			61,608 (21.66)		28,019 (22.85)		5220 (28.93)		4149 (27.52)		37,388 (24)
**Drinking frequency, n (%)**												
	Never			163,840 (57.59)		63,675 (51.92)		9844 (54.56)		7381 (48.96)		80,900 (51.94)
	2-3 per month			44,889 (15.78)		17,217 (14.04)		2505 (13.88)		1888 (12.52)		21,610 (13.87)
	1-4 per week			60,578 (21.29)		32,324 (26.36)		4379 (24.27)		4389 (29.11)		41,092 (26.38)
	Every day			9929 (3.49)		7219 (5.89)		941 (5.22)		1134 (7.52)		9294 (5.97)
**Exercise frequency, n (%)**												
	Never			158,372 (55.67)		68,534 (55.88)		9959 (55.2)		8297 (55.03)		86,790 (55.72)
	1-4 times per week			93,821 (32.98)		38,416 (31.32)		5711 (31.65)		4643 (30.8)		48,770 (31.31)
	5-7 times per week			24,834 (8.73)		12,048 (9.82)		1934 (10.72)		1778 (11.79)		15,760 (10.12)
**Charlson Comorbidity Index scores, n (%)**												
	0			146,346 (51.44)		61,699 (50.31)		6616 (36.67)		5931 (39.34)		74,246 (47.67)
	1			86,439 (30.38)		36,653 (29.89)		4919 (27.26)		4092 (27.14)		45,664 (29.32)
	2			34,140 (12)		15,385 (12.54)		2878 (15.95)		2360 (15.65)		20,623 (13.24)
	≥3			17,559 (6.17)		8909 (7.26)		3629 (20.11)		2694 (17.87)		15,232 (9.78)
**Disability severity, n (%)**												
	No disability			283,347 (99.6)		121,870 (99.37)		17,914 (99.29)		14,952 (99.17)		154,736 (99.34)
	Mild			728 (0.26)		499 (0.41)		66 (0.37)		65 (0.43)		630 (0.4)
	Severe			409 (0.14)		277 (0.23)		62 (0.34)		60 (0.40)		399 (0.26)
Number of outpatient visits and hospitalization days, mean (SD)				14.2 (12.6)		16.6 (14.13)		20.0 (15.65)		20.4 (15.7)		17.3 (14.5)
**Use of medication, n (%)**												
	Yes			15,245 (5.36)		24,226 (19.75)		5939 (32.92)		6027 (39.97)		36,192 (23.24)
	Antidiabetic medication			3200 (1.12)		2841 (2.32)		5126 (28.41)		4262 (28.27)		12,229 (7.85)
	Antihypertensive medication			13,129 (4.62)		23,169 (18.89)		2205 (12.22)		4149 (27.52)		29,523 (18.95)
	Antidiabetic and antihypertensive medication			1084 (0.38)		1784 (1.45)		1392 (7.72)		2384 (15.81)		5560 (3.57)
**Use of antidiabetic medication, n (%)**												
	Yes			3200 (1.12)		2841 (2.32)		5126 (28.41)		4262 (28.27)		12,229 (7.85)
	**MPR^b^, n/N (%)**											
		<70%	1210/3200 (37.81)		1028/2841 (36.18)		2042/5126 (39.84)		1714/4262 (40.22)		4784/12,229 (39.12)	
		≥70%	1990/3200 (62.19)		1813/2841 (63.82)		3084/5126 (60.16)		2548/4262 (59.78)		7445/12,229 (60.88)	
**Use of antihypertensive medication, n (%)**												
	Yes			13,129 (4.62)		23,169 (18.89)		2205 (12.22)		4149 (27.52)		29,523 (18.95)
	**MPR, n/N (%)**											
		<70%	4404/13,129 (33.54)		8066/23,169 (34.81)		774/2205 (35.1)		1468/4149 (35.38)		10,308/29,523 (34.92)	
		≥70%	8725/13,129 (66.46)		15,103/23,169 (65.19)		1431/2205 (64.9)		2681/4149 (64.62)		19,215/29,523 (65.08)	
**Use of antihyperlipidemic medication, n (%)**												
	Yes			2839 (1)		2569 (2.09)		818 (4.53)		742 (4.92)		4129 (2.65)
	**MPR, n/N (%)**											
		<70%	1181/2839 (41.6)		1050/2569 (40.87)		343/818 (41.93)		334/742 (45.01)		1727/4129 (41.83)	
		≥70%	1658/2839 (58.4)		1519/2569 (59.13)		475/818 (58.07)		408/742 (54.99)		2402/4129 (58.17)	

^a^Except for the mean medication possession ratio, all variables significantly differed between the well-controlled group and the total uncontrolled groups at *P*<.001.

^b^MPR: medication possession ratio.

### Incidence of MACE or Death From Any Cause

**Figure 2:**
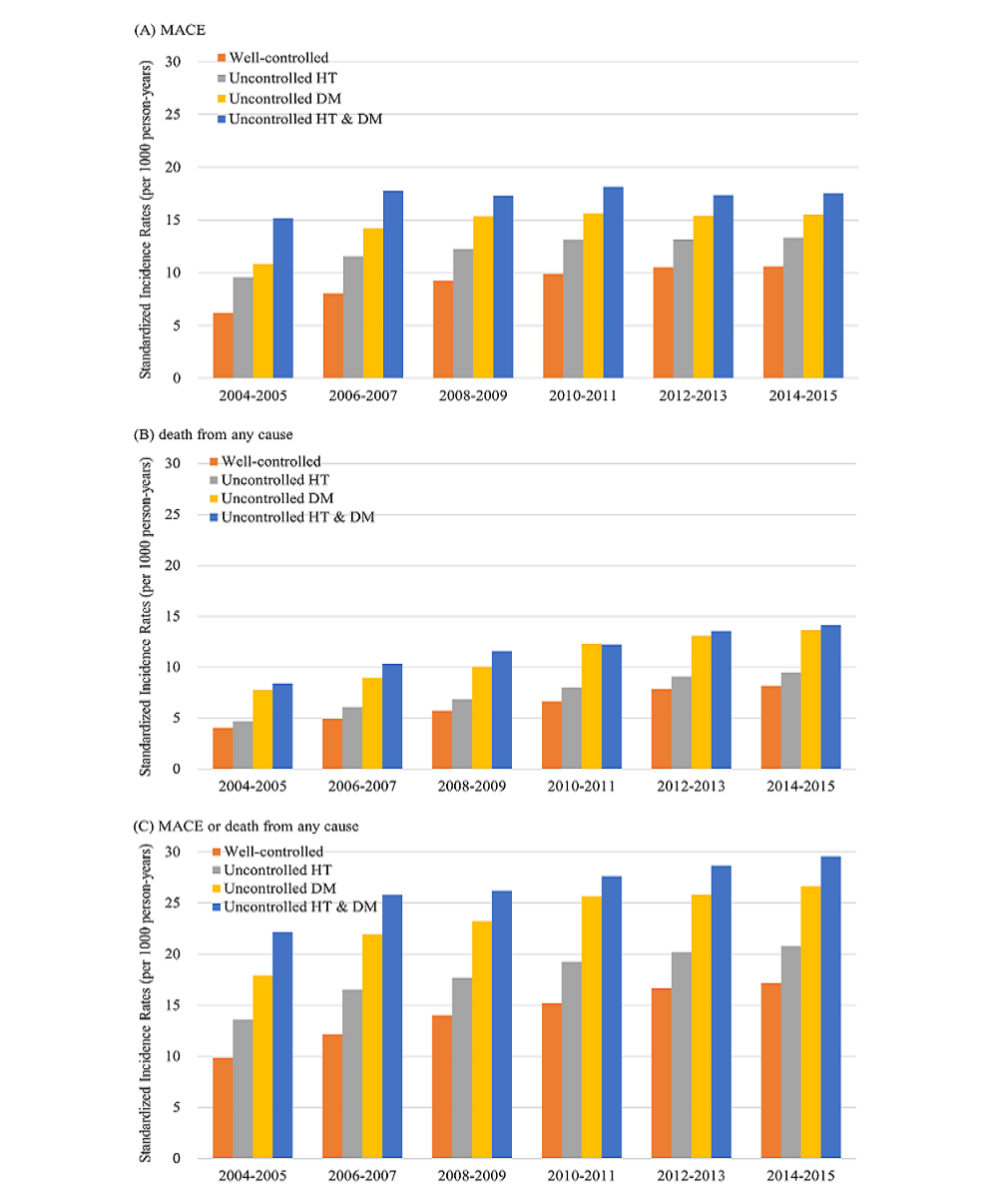
(A) Incidence rates of major adverse cardiovascular event (MACE); (B) death from any cause; and (C) MACE or death from any cause per 1000 person-years by the control status of hypertension (HT) or diabetes mellitus (DM).

The incidence of MACE increased in the order of the uncontrolled HT group, uncontrolled DM group, and uncontrolled HT and DM group (Figure 2A; Table S2 in Multimedia Appendix 1). Moreover, during the follow-up periods from index periods 1 to 6 with increasing age, the incidence continued to grow; the absolute change in the incidence of MACE was +4.4 (range 6.2-10.6), +3.7 (range 9.6-13.3), and +4.7 (range 10.8-15.5) cases per 1000 person-years among the well-controlled group, uncontrolled HT group, and uncontrolled DM group, respectively. However, in the uncontrolled HT and DM group, the incidence of MACE was high at a relatively young age (15.2 at the age of 52 years on average in 2004-2005 and 17.5 at the age of 61 years on average in 2014-2015) and showed no increasing trend (15.2, 17.8, 17.3, 18.1, 17.3, and 17.5 at every 2 years from 2004-2005 to 2014-2015).

With respect to death from any cause, the incidence rates per 1000 person-years were higher in the uncontrolled group than in the well-controlled group, especially in the uncontrolled DM group (Figure 2B; Table S2 in Multimedia Appendix 1). In all groups, the increase with age was more pronounced in deaths from any cause than in MACE. Between the follow-up periods 1 and 6, the absolute change in the incidence of death from any cause was +4.1 (range 4.0-8.2), +4.8 (range 4.7-9.5), +5.9 (range 7.8-13.7), and +5.7 (range 8.4-14.1) cases per 1000 person-years among the well-controlled group, the uncontrolled HT group, uncontrolled DM group, and the uncontrolled HT and DM group, respectively.

During each follow-up period, the incidence rates of MACE or death from any cause in the uncontrolled HT and DM group were approximately twice of that in the well-controlled group. The incidence of MACE or death from any cause increased with age (Figure 2C; Table S2 in Multimedia Appendix 1).

### Risk of MACE or Death From Any Cause

Univariable analyses showed that higher age, lower income level, poor adherence (MPR <70%), poor health behaviors (underweight or obese, smoking, drinking every day, and no exercising), or more frequent outpatient visits and hospital stays may be associated with an increased cardiovascular or mortality risk. These variables with statistically significant association on univariable analyses were included in the multivariable model as time-varying covariates for MACE and death from any cause (Table S3 in Multimedia Appendix 1). Uncontrolled DM or HT status was associated with an increased risk of MACE and all-cause mortality (Table 2). Compared with the well-controlled group, the event-rate increase of MACE was 28% in the uncontrolled DM group (HR 1.28, 95% CI 1.23-1.32), 32% in the uncontrolled HT group (HR 1.32, 95% CI 1.29-1.35), and 54% in the uncontrolled HT and DM group (HR 1.54, 95% CI 1.47-1.60). With respect to death from any cause, the event-rate increase was 5% in the uncontrolled DM group (HR 1.05, 95% CI 1.01-1.10), 13% in the uncontrolled HT group (HR 1.13, 95% CI 1.10-1.16), and 17% in the uncontrolled HT and DM group (HR 1.17, 95% CI 1.12-1.23).

**Table 2:** Adjusted hazard ratios (HRs) for major adverse cardiovascular event (MACE), death from any cause, and MACE or death from any cause associated with risk factors (multivariable analysis^a^).

Control status of HT^b^ or DM^c^	MACE		Death from any cause		MACE or death from any cause	
	HR (95% CI)	*P* value	HR (95% CI)	*P* value	HR (95% CI)	*P* value
Well-controlled	1.00	Reference	1.00	Reference	1.00	Reference
Uncontrolled HT	1.32 (1.29-1.35)	<.001	1.13 (1.10-1.16)	<.001	1.24 (1.22-1.27)	<.001
Uncontrolled DM	1.28 (1.23-1.32)	<.001	1.05 (1.01-1.10)	<.001	1.18 (1.14-1.21)	<.001
Uncontrolled HT and DM	1.54 (1.47-1.60)	<.001	1.17 (1.12-1.23)	<.001	1.36 (1.31-1.40)	<.001

^a^The analysis based on extended Cox models was adjusted for time-dependent variables, such as age, sex, type of national health security program, income level, BMI, smoking, drinking frequency, exercise frequency, Charlson Comorbidity Index scores, disability severity, number of outpatient visits and hospitalization days, and medication possession ratio.

^b^HT: hypertension.

^c^DM: diabetes mellitus.

## Discussion

### Principal Findings

This is the first nationwide retrospective cohort study to explore the latest incidence of MACE or death from any cause by HT or DM control status and their association, reflecting the time-varying sociodemographic and clinical characteristics of the population in South Korea.

We observed that 35.38% (155,765/440,249) of the total cohort aged 40 to 79 years belonged to the uncontrolled group for HT or DM in the first index period, and the proportion of uncontrolled HT or DM appeared to decrease over time. However, this decreasing trend may be explained by the exclusion of uncontrolled patients who died or developed MACE over time in the cohort setting. Medication use is important for BP or FBG control [7,10]. Approximately 23.24% (36,192/155,765) of the patients with uncontrolled DM or HT were prescribed medication. Similar to our study, previous studies have shown low prescription rates in patients with HT and DM [3]. Among medication users, uncontrolled HT or DM accounted for >60% of patients, which was comparable with the BP and blood glucose control rate of 50% for the treated patients reported in foreign real-world clinical practice [21,22]. These results may be because possessing a prescribed medication does not necessarily mean adherence to the treatment. Nonetheless, our findings emphasize the need for novel BP- and blood glucose–lowering strategies, along with managing modifiable risk factors for cardiovascular diseases. The implementation of an efficient system such as mobile health, which has been proven effective in controlling HT and DM by continuously monitoring diseases through improving the health care delivery process, may be considered [23,24]. Along with this, sustained, community-wide programs targeting cardiovascular risk factors and behavioral changes to improve health would be beneficial [5].

Our study also showed the association between medication use and MACE or death from any cause (Table S3 in Multimedia Appendix 1). The risk of MACE or death from any cause was higher in the poor adherence group (MPR <70%) than in the adherence group (MPR ≥70%). It is well known that poor adherence to antihypertensive medication and antidiabetic medication may increase the risk of cardiovascular events [25,26]. Therefore, understanding the factors related to adherence to medication could be the key to preventing MACE and death. Strategies tailored to the economical, geographical, sociological, educational, and health care system contexts of patients are effective in improving adherence to medication [27]. Further studies are needed to identify the barriers that affect adherence to medication. In addition, the study showed that nonmedication users may have a lower risk of MACE or death from any cause than medication users. A lower risk for nonmedication users was expected because most of them achieved controlled BP or glycemic status without medication use. According to the guidelines on the prevention of cardiovascular diseases, patients with HT or DM requiring medication treatment have a higher cardiovascular risk than those who do not require medication treatment [7].

The study indicated that poor health behaviors (underweight or obese, smoking, drinking every day, and no exercise) or more frequent outpatient visits and hospital stays may be associated with an increased cardiovascular or mortality risk. These findings are consistent with previously reported results [28]. In terms of drinking behavior, a trend toward higher mortality or cardiovascular risk was observed in nondrinkers than in moderate drinkers. The causal role of alcohol consumption in cardiovascular diseases remains unclear [29].

We found that the incidence of MACE in South Korean adults aged 40 to 79 years was 10.8 to 15.5 and 9.6 to 13.3 per 1000 person-years during the follow-up periods in the uncontrolled DM and HT groups, respectively. After adjusting for medication use and other risks, the uncontrolled HT and DM groups had a 1.32-fold and 1.28-fold higher risk of MACE than the well-controlled group, respectively. In particular, the uncontrolled HT and DM group had a 1.54-fold higher risk of MACE than the well-controlled group. The higher incidence in the uncontrolled group may have been influenced by the relatively poor health behaviors and low economic status of the uncontrolled group. These clustered factors are likely to mediate the impact on ill health and premature mortality [30] and interact synergistically, increasing the individual’s total risk of cardiovascular diseases [4]. Most previous studies have estimated the incidence of cardiovascular disease in newly diagnosed patients, regardless of the control status of BP or FBG [2,31]. In an earlier study including patients in South Korea aged 20 to 89 years with type 2 diabetes, the incidence of hospitalization for coronary artery disease and cerebrovascular disease per 1000 person-years was reported as 6.11 and 4.50, respectively, during 2009 to 2011. These results, which are lower than those in our study, could be because the study was conducted on patients with DM who have prescribed medication and included some patients with controlled DM.

In this study, the patients were older, and the mean age of the total cohort from 2002 to 2003 was 52 years, and the mean age of the cohort in 2012 to 2013, excluding patients in whom the outcomes occurred, was 61 years. Similar to a previous study [32], the incidence of MACE tended to increase with age in the uncontrolled HT group and uncontrolled DM group. This trend was particularly marked for the incidence of death from any cause. However, in the uncontrolled HT and DM group, the incidence of MACE was high at a relatively early age, and no age-related trends were observed. There were quite a few participants in the uncontrolled HT and DM group in the study, accounting for 9.68% (15,077/155,765) of the total uncontrolled group. The percentage of uncontrolled HT and DM group may be underestimated owing to the masking HT in patients with high FBG levels [33,34]. HT and DM are common comorbidities linked by various vascular mechanisms and risk factors and accelerate each other’s development [35]. The coexistence of HT and DM contributes to the development of cardiovascular disease by amplifying vascular damage and endothelial dysfunction [36]. Therefore, managing these patients at a young age can substantially reduce the incidence of cardiovascular diseases. For example, if patients with uncontrolled HT and DM control their BP and FBG levels at the age of 52 years, the incidence of MACE would be reduced by 60% (6.2 and 15.2 per 1000 person-years in the well-controlled group and the uncontrolled HT and DM group, respectively; Table S2 in Multimedia Appendix 1). These findings indicate that the uncontrolled HT and DM group requires strict management as early as possible. For example, more aggressive treatment with a BP target of <130/80 mm Hg could be considered in adults with DM and HT, consistent with the American Society’s recommendation for managing high BP [37].

In traditional Cox regression models, risk factors measured at baseline are usually associated with clinical outcomes occurring after a period. However, things may change during follow-up; either the effect of a fixed baseline risk factor may vary over time, resulting in a weakening or strengthening of associations over time, or the risk factor itself may vary over time [38]. Thus, a more detailed analysis of lifetime risks would take repeated measures into consideration for potential changes and would adjust for the time-dependent effect of contributing risk factors [39]. Lifestyle factors such as medication use, smoking, exercise, and obesity have changed significantly over the past 20 years among adults in South Korea [6], and BP and FBG levels are also factors that change over time. However, few studies have been conducted taking this into account to examine the association of control of HT or DM with cardiovascular events or mortality. This study accounted for changes in these factors over time. A previous study using traditional Cox analysis conducted in South Korea found that HT and DM were more strongly associated with hospitalization for coronary heart disease than that reported in our results [40]. The conservative results in our study may be explained by the fact that most important clinical risk factors such as medication prescription and adherence were considered and reflected as time-dependent manners. Our results using extended Cox analysis with adjusting various time-dependent factors indicated that uncontrolled HT and uncontrolled DM were independent risk factors for MACE.

This study has several strengths. First, the study used a large national cohort with a low rate of follow-up loss over a period of >10 years [17]. Second, when potential risk factors change over time, it may be inappropriate from a clinical perspective to correlate all future survival rates with the factors evaluated at one moment [38]. Thus, time-varying variables such as BP, FBG, medication prescription, and adherence were adjusted using extended Cox analysis. It allows for the accurate attribution of risk factors for each patient at the time of MACE or death events. Third, as clinical laboratory tests were performed for all national health screening program participants, our study included not only diagnosed patients but also patients who were not aware of HT or DM or were diagnosed but untreated patients. Diagnosis based on these clinical data decreases the likelihood of misclassification of disease status, which causes a bias of estimates of association [41].

### Limitations

This study had some limitations. First, BP was measured using a device validated in various hospitals and clinics certified by the National Health Insurance Service. However, the BP measurement devices used at each institution were not uniform, and the measured values may differ depending on the measurement method. BP measured using automated office BP measurements is generally 5 to 10 mm Hg lower than the typical manual office BP [42,43]. Second, we used FBG criteria for the diagnosis of DM, not hemoglobin A_1c_ (HbA_1c_) criteria, because the NHIS-HEALS database did not include the HbA_1c_ variable. However, FBG levels were closely associated with specific HbA_1c_ levels in the South Korean population, and FBG criteria were reported to be valid in the South Korean population [44]. Third, although our study controlled for various measured confounders, including medication use and patient characteristics by multivariable analysis, additional or unmeasured confounders may exist. Fourth, as our data were not linked to the cause of death data provided by Statistics Korea, we could not estimate the cause-specific mortality. Fifth, although we considered time-varying cardiovascular risk profiles and lifestyle using extended Cox analysis, the trajectories of BP or FBG changes over the observation period were not reflected. Trajectory modeling assessing the association of health outcomes with subgroups of individuals that share similar patterns of laboratory assessments is a subject worthy of further research [45]. Despite these limitations, the screening cohort data, including laboratory results and all health care use information used in this study, provide a powerful resource for an up-to-date population-based evaluation.

### Conclusions

This study highlights the importance of controlling BP or FBG levels to reduce the risk of MACE or death. This up-to-date understanding of the association between BP and FBG control and cardiovascular events serves as the basic data for planning public health policies to prevent cardiovascular diseases. Moreover, the high uncontrolled rates of HT or DM, regardless of medication prescription, have led us to suggest the need for novel efficient systems such as a community-wide management program using mobile health technology that is effective in continuously controlling high BP and blood glucose levels.

## Appendix

Multimedia Appendix 1 Selection flowchart of study population, definition of outcomes, and incidence rates and hazard ratios of major adverse cardiovascular events (MACE), death from any cause, and MACE or death from any cause.

## Abbreviations

BP: blood pressure
CCI: Charlson Comorbidity Index
DM: diabetes mellitus
FBG: fasting blood glucose
HbA_1c_: hemoglobin A_1c_
HR: hazard ratio
HT: hypertension
IHD: ischemic heart disease
MACE: major adverse cardiovascular events
MPR: medication possession ratio
NHIS-HEALS: National Health Insurance Service–National Health Screening Cohort

## Data Availability

The data underlying the results presented in the study are available from the National Health Insurance Data Sharing Service after the committee of the National Health Insurance Service reviews the application for approval of the use of National Health Insurance Service–National Health Screening Cohort data. Researchers receive access privileges to the requested database after paying the fee. Interested researchers will be able to access the data in the same manner and replicate the findings of our study by following the protocol outlined in the *Methods* section.
