# Supplementary material for: Association of Uncontrolled Hypertension or Diabetes Mellitus With Major Adverse Cardiovascular Events and Mortality in South Korea: Population-Based Cohort Study
Source: JMIR Public Health Surveill. 2023 Feb 3;9:e42190. doi: 10.2196/42190 (PMC9938442; doi:10.2196/42190)
Supplement: Multimedia Appendix 1 [file publichealth_v9i1e42190_app1.docx]

**Multimedia Appendix 1**

**
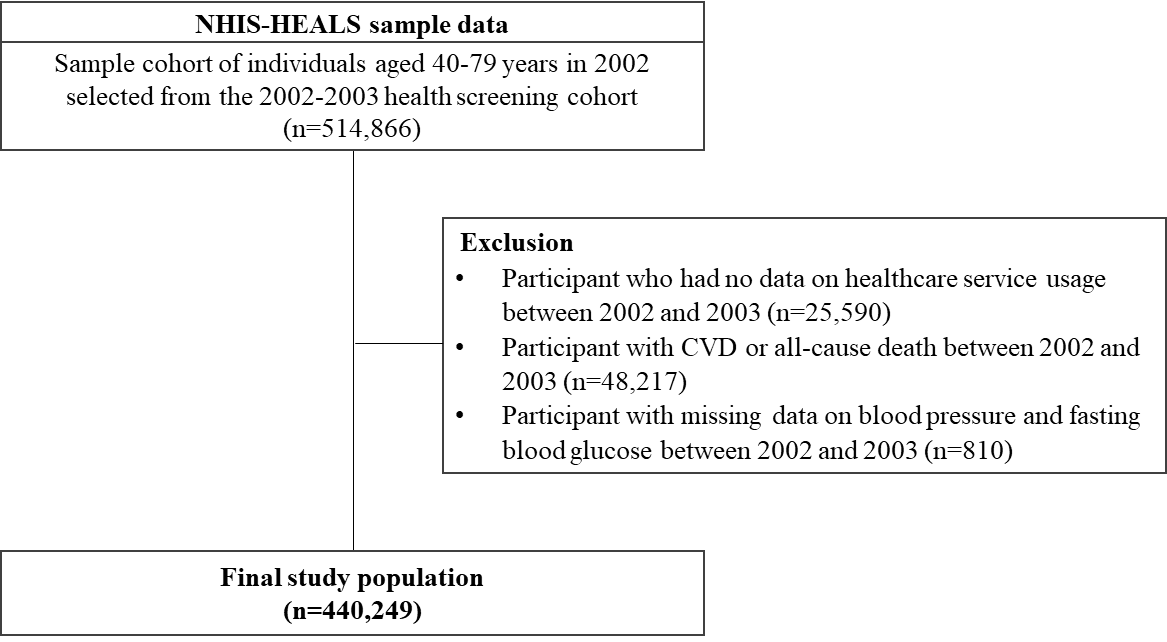
**

**Figure S1. Selection flowchart of study population.**

Table S1: Definition of Outcomes.

| Major adverse cardiovascular events | | ICD-10^a^ codes |
| --- | --- | --- |
| Ischaemic heart diseases | Angina pectoris | I20 |
|  | Myocardial infarction | I21, I22, I23 |
|  | Other acute ischaemic heart diseases | I24 |
|  | Chronic ischaemic heart disease | I25 |
| Cerebrovascular diseases | Transient cerebral ischaemic attacks and related syndromes | G45 |
|  | hemorrhagic stroke | I60-I62 |
|  | Cerebral infarction | I63 |
|  | Stroke, not specified as haemorrhage or infarction | I64 |
|  | Other cerebrovascular diseases | I65-I69 |
| Heart failure | Heart failure | I50, I11.0, I13.0, I13.2 |
| ^a^ICD-10, International Classification of Diseases, 10th revision | |  |

Table S2: Incidence rates of MACE, Death from any cause, and MACE or death from any cause per 1000 Person-years by the control status of HT or DM.

| Follow-up period |  | MACE^a^ | | | | | Death from any cause | | | | | MACE or death from any cause | | | | | |
| --- | --- | --- | --- | --- | --- | --- | --- | --- | --- | --- | --- | --- | --- | --- | --- | --- | --- |
|  |  | All | Well-controlled | Uncontrolled | | | All | Well-controlled | Uncontrolled | | | All | Well-controlled | Uncontrolled | | |  |
|  |  |  |  | HT^b^ | DM^c^ | HT & DM |  |  | HT | DM | HT & DM |  |  | HT | DM | HT & DM |  |
| 2004-2005 | n | 440,249 | 284,484 | 122,646 | 18,042 | 15,077 | 440,249 | 284,484 | 122,646 | 18,042 | 15,077 | 440,249 | 284,484 | 122,646 | 18,042 | 15,077 |  |
|  | Person-Years | 873,859 | 566,107 | 242,569 | 35,638 | \| 29,546 \| 876686 \| 567125 \| \| --- \| --- \| --- \| | 876,686 | 567,125 | 243,969 | 35,768 | 29,823 | 870,457 | 564,411 | 241,428 | 35,352 | 29,265 |  |
|  | Events | 6,961 (1.6%) | 3,074 (1.1%) | 2,819 (2.3%) | 452 (2.5%) | 616 (4.1%) | 4,079 (0.9%) | 1,941 (0.7%) | 1,440 (1.2%) | 333 (1.8%) | 365 (2.4%) | 10,550 (2.4%) | 4,847 (1.7%) | 4,041 (3.3%) | 749 (4.2%) | 913 (6.1%) |  |
|  | Incidence Rates^d^ | 8.0 | 6.2 | 9.6 | 10.8 | 15.2 | 4.7 | 4.0 | 4.7 | 7.8 | 8.4 | 12.1 | 9.9 | 13.6 | 17.9 | 22.2 |  |
| 2006-2007 | n | 433,288 | 290,899 | 109,752 | 18,993 | 13,644 | 436,170 | 292,246 | 110,929 | 19,135 | 13,860 | 429,699 | 289,100 | 108,559 | 18,698 | 13,342 |  |
|  | Person-Years | 857,031 | 576,862 | 216,215 | 37,326 | 26,629 | 866,401 | 581,347 | 219,892 | 37,842 | 27,320 | 846,070 | 571,322 | 212,548 | 36,426 | 25,774 |  |
|  | Events | 8,416 (1.9%) | 4,132 (1.4%) | 3,043 (2.8%) | 610 (3.2%) | 631 (4.6%) | 4,981 (1.1%) | 2,426 (0.8%) | 1,745 (1.6%) | 400 (2.1%) | 410 (3.0%) | 12,346 (2.9%) | 6,132 (2.1%) | 4,375 (4.0%) | 929 (5.0%) | 910 (6.8%) |  |
|  | Incidence Rates | 9.8 | 8.0 | 11.6 | 14.2 | 17.8 | 5.7 | 4.9 | 6.1 | 8.9 | 10.3 | 14.6 | 12.2 | 16.5 | 22.0 | 25.8 |  |
| 2008-2009 | n | 424,872 | 299,722 | 91,988 | 21,018 | 12,144 | 431,189 | 303,441 | 93,743 | 21,541 | 12,464 | 417,353 | 295,859 | 89,541 | 20,397 | 11,556 |  |
|  | Person-Years | 840,811 | 594,485 | 181,242 | 41,337 | 23,746 | 856,850 | 603,993 | 185,757 | 42,573 | 24,528 | 821,643 | 584,462 | 175,115 | 39,737 | 22,330 |  |
|  | Events | 8,976 (2.1%) | 5,010 (1.7%) | 2,722 (3.0%) | 708 (3.4%) | 536 (4.4%) | 5,595 (1.3%) | 2,963 (1.0%) | 1,727 (1.8%) | 490 (2.3%) | 415 (3.3%) | 13,129 (3.1%) | 7,363 (2.5%) | 3,937 (4.4%) | 1,042 (5.1%) | 787 (6.8%) |  |
|  | Incidence Rates | 10.7 | 9.3 | 12.2 | 15.3 | 17.3 | 6.5 | 5.7 | 6.8 | 10.0 | 11.6 | 16.0 | 14.0 | 17.7 | 23.2 | 26.2 |  |
| 2010-2011 | n | 415,896 | 302,953 | 78,012 | 24,021 | 10,910 | 425,594 | 309,569 | 79,842 | 24,984 | 11,199 | 404,224 | 296,677 | 74,435 | 23,034 | 10,078 |  |
|  | Person-Years | 821,485 | 599,625 | 153,387 | 47,165 | 21,308 | 843,731 | 614,757 | 157,743 | 49,246 | 21,985 | 793,796 | 584,488 | 145,142 | 44,739 | 19,427 |  |
|  | Events | 9,230 (2.2%) | 5,524 (1.8%) | 2,422 (3.1%) | 801 (3.3%) | 483 (4.4%) | 6,384 (1.5%) | 3,585 (1.2%) | 1,739 (2.2%) | 677 (2.7%) | 383 (3.4%) | 13,640 (3.4%) | 8,152 (2.7%) | 3,527 (4.7%) | 1,265 (5.5%) | 696 (6.9%) |  |
|  | Incidence Rates | 11.2 | 9.9 | 13.1 | 15.6 | 18.1 | 7.6 | 6.6 | 8.0 | 12.3 | 12.2 | 17.2 | 15.2 | 19.3 | 25.7 | 27.6 |  |
| 2012-2013 | n | 406,666 | 298,132 | 71,761 | 25,910 | 10,863 | 419,210 | 307,224 | 73,690 | 27,114 | 11,182 | 390,584 | 289,229 | 67,100 | 24,445 | 9,810 |  |
|  | Person-Years | 803,784 | 590,358 | 141,221 | 50,929 | 21,277 | 831,243 | 610,292 | 145,553 | 53,487 | 21,911 | 766,817 | 569,582 | 130,779 | 47,545 | 18,912 |  |
|  | Events | 9,323 (2.3%) | 5,764 (1.9%) | 2,244 (3.1%) | 885 (3.4%) | 430 (4.0%) | 7,251 (1.7%) | 4,194 (1.4%) | 1,860 (2.5%) | 744 (2.7%) | 453 (4.1%) | 14,136 (3.6%) | 8,721 (3.0%) | 3,388 (5.0%) | 1,338 (5.5%) | 689 (7.0%) |  |
|  | Incidence Rates | 11.6 | 10.5 | 13.2 | 15.4 | 17.3 | 8.7 | 7.8 | 9.1 | 13.1 | 13.5 | 18.4 | 16.7 | 20.2 | 25.9 | 28.7 |  |
| 2014-2015 | n | 397,343 | 290,932 | 68,018 | 27,703 | 10,690 | 411,959 | 301,797 | 69,846 | 29,353 | 10,963 | 376,448 | 279,102 | 62,211 | 25,767 | 9,368 |  |
|  | Person-Years | 784,396 | 575,302 | 133,741 | 54,450 | 20,902 | 815,427 | 598,535 | 137,621 | 57,791 | 21,480 | 737,878 | 548,747 | 121,037 | 50,062 | 18,032 |  |
|  | Events | 9,186 (2.3%) | 5,786 (2.0%) | 2,084 (3.1%) | 884 (3.2%) | 432 (4.0%) | 7,427 (1.8%) | 4,304 (1.4%) | 1,869 (2.7%) | 836 (2.8%) | 418 (3.8%) | 14,031 (3.7%) | 8,749 (3.1%) | 3,211 (5.2%) | 1,404 (5.4%) | 667 (7.1%) |  |
|  | Incidence Rates | 11.7 | 10.6 | 13.3 | 15.5 | 17.5 | 9.1 | 8.2 | 9.5 | 13.7 | 14.1 | 19.0 | 17.2 | 20.8 | 26.7 | 29.6 |  |

^a^major adverse cardiovascular event.

^b^hypertension.

^c^diabetes mellitus.

^d^The rates were standardized to the age distribution from each time period.

Table S3: Hazard Ratios for MACE, Death from any cause, and MACE or death from any cause associated with risk factors (Univariable analyses).

| Variables | MACE^a^ | | | Death from any cause | | | MACE or death from any cause | | |
| --- | --- | --- | --- | --- | --- | --- | --- | --- | --- |
|  | HR^b^ | (95% CI^c^) | *P* value | HR | (95% CI) | *P* value | HR | (95% CI) | *P* value |
| **Control status of HT^d^ or DM^e^** | | | | | | | | | |
| Well-controlled | 1.00 |  |  | 1.00 |  |  | 1.00 |  |  |
| Uncontrolled HT | 1.75 | (1.72-1.79) | <.001 | 1.83 | (1.79-1.88) | <.001 | 1.75 | (1.72-1.78) |  |
| Uncontrolled DM | 1.89 | (1.83-1.95) | <.001 | 2.20 | (2.12-2.28) | <.001 | 1.98 | (1.93-2.03) |  |
| Uncontrolled HT & DM | 2.59 | (2.49-2.69) | <.001 | 3.08 | (2.95-3.22) | <.001 | 2.69 | (2.61-2.78) |  |
| **Use of antidiabetic medication** | | | | | | | | | |
| MPR^f^ = 0^g^ | 0.47 | (0.46-0.49) | <.001 | 0.43 | (0.42-0.45) | <.001 | 0.47 | (0.46-0.48) | <.001 |
| MPR <70 | 1.53 | (1.44-1.62) | <.001 | 5.14 | (4.91-5.37) | <.001 | 2.68 | (2.58-2.78) | <.001 |
| MPR ≥70 | 1.00 |  |  | 1.00 |  |  | 1.00 |  |  |
| **Use of antihypertensive medication** | | | | | | | | | |
| MPR = 0^g^ | 0.48 | (0.47-0.49) | <.001 | 0.43 | (0.42-0.45) | <.001 | 0.50 | (0.49-0.51) | <.001 |
| MPR <70 | 1.64 | (1.58-1.70) | <.001 | 5.42 | (5.26-5.59) | <.001 | 2.78 | (2.71-2.85) | <.001 |
| MPR ≥70 | 1.00 |  |  | 1.00 |  |  | 1.00 |  |  |
| **Use of hyperlipidemia medication** | | | | | | | | | |
| MPR =0^g^ | 0.59 | (0.57-0.60) | <.001 | 0.67 | (0.64-0.69) | <.001 | 0.69 | (0.67-0.71) | <.001 |
| MPR <70 | 1.47 | (1.40-1.56) | <.001 | 4.96 | (4.74-5.19) | <.001 | 2.43 | (2.33-2.53) | <.001 |
| MPR ≥70 | 1.00 |  |  | 1.00 |  |  | 1.00 |  |  |
| **Age** | | | | | | | | | |
| 40–<65 | 1.00 |  |  | 1.00 |  |  | 1.00 |  |  |
| 65–<70 | 2.33 | (2.27-2.39) | <.001 | 3.43 | (3.31-3.54) |  | 2.59 | (2.54-2.64) | <.001 |
| 70–<80 | 3.34 | (3.27-3.42) | <.001 | 7.88 | (7.67-8.09) |  | 4.38 | (4.30-4.46) | <.001 |
| ≥80 | 4.04 | (3.87-4.22) | <.001 | 21.79 | (21.04-22.57) |  | 8.55 | (8.30-8.80) | <.001 |
| **Sex** | | | | | | | | | |
| Male | 1.00 |  |  | 1.00 |  |  | 1.00 |  |  |
| Female | 0.89 | (0.87-0.91) | <.001 | 0.53 | (0.52-0.54) |  | 0.72 | (0.71-0.73) | <.001 |
| **Income level** | | | | | | | | | |
| Lowest | 1.25 | (1.22-1.28) | <.001 | 1.76 | (1.71-1.82) | <.001 | 1.42 | (1.39-1.45) | <.001 |
| Low–middle | 1.10 | (1.07-1.13) | <.001 | 1.41 | (1.37-1.45) | <.001 | 1.22 | (1.19-1.24) | <.001 |
| Middle–high | 1.05 | (1.03-1.07) | <.001 | 1.18 | (1.14-1.21) | <.001 | 1.11 | (1.09-1.13) | <.001 |
| Highest | 1.00 |  |  | 1.00 |  |  | 1.00 |  |  |
| **Type of national health security program** | | | | | | | | | |
| National Health Insurance | 1.00 |  |  | 1.00 |  |  | 1.00 |  |  |
| Medical Aid | 2.16 | (2.04-2.29) | <.001 | 3.42 | (3.24-3.62) | <.001 | 2.66 | (2.55-2.79) | <.001 |
| **Body Mass Index (kg/m^2^)** | | | | | | | | | |
| <18.5 | 1.24 | (1.17-1.30) | <.001 | 3.47 | (3.34-3.61) | <.001 | 2.11 | (2.04-2.19) | <.001 |
| ≥18.5–<25 | 1.00 |  |  | 1.00 |  |  | 1.00 |  |  |
| ≥25–<30 | 1.19 | (1.17-1.21) | <.001 | 0.73 | (0.71-0.74) | <.001 | 1.02 | (1.00-1.04) | 0.02 |
| ≥30 | 1.31 | (1.25-1.38) | <.001 | 0.77 | (0.71-0.83) | <.001 | 1.12 | (1.07-1.17) | <.001 |
| **Smoking** | | | | | | | | | |
| Non-­smoker | 1.00 |  |  | 1.00 |  |  | 1.00 |  |  |
| Ex­-smoker | 1.01 | (0.98-1.03) | 0.66 | 1.15 | (1.11-1.18) | <.001 | 1.08 | (1.06-1.11) | <.001 |
| Current smoker | 1.18 | (1.15-1.21) | <.001 | 1.66 | (1.61-1.70) | <.001 | 1.39 | (1.36-1.41) | <.001 |
| **Drinking frequency** | | | | | | | | | |
| Never | 1.00 |  |  | 1.00 |  |  | 1.00 |  |  |
| 2–3 per month | 0.75 | (0.73-0.77) | <.001 | 0.59 | (0.57-0.61) | <.001 | 0.71 | (0.70-0.73) | <.001 |
| 1–4 per week | 0.83 | (0.81-0.84) | <.001 | 0.80 | (0.78-0.83) | <.001 | 0.85 | (0.83-0.86) | <.001 |
| Everyday | 1.11 | (1.06-1.16) | <.001 | 1.89 | (1.81-1.96) | <.001 | 1.46 | (1.42-1.51) | <.001 |
| **Exercise frequency** | | | | | | | | | |
| Never | 1.00 |  |  | 1.00 |  |  | 1.00 |  |  |
| 1–4 times per week | 0.74 | (0.73-0.76) | <.001 | 0.51 | (0.50-0.53) | <.001 | 0.66 | (0.65-0.67) | <.001 |
| 5–7 times per week | 0.85 | (0.83-0.87) | <.001 | 0.53 | (0.51-0.54) | <.001 | 0.73 | (0.71-0.74) | <.001 |
| **Charlson Comorbidity Index scores** | | | | | | | | | |
| 0 | 1.00 |  |  | 1.00 |  |  | 1.00 |  |  |
| 1 | 1.46 | (1.42-1.50) | <.001 | 1.37 | (1.32-1.43) | <.001 | 1.38 | (1.35-1.41) | <.001 |
| 2 | 2.01 | (1.95-2.06) | <.001 | 2.15 | (2.07-2.23) | <.001 | 1.95 | (1.91-2.00) | <.001 |
| ≥3 | 3.10 | (3.03-3.18) | <.001 | 7.32 | (7.10-7.56) | <.001 | 4.04 | (3.96-4.12) | <.001 |
| **Disability severity** | | | | | | | | | |
| No disability | 1.00 |  |  | 1.00 |  |  | 1.00 |  |  |
| Severe | 2.23 | (1.98-2.51) | <.001 | 6.60 | (6.06-7.19) | <.001 | 3.64 | (3.36-3.96) | <.001 |
| Mild | 2.35 | (2.16-2.55) | <.001 | 5.56 | (5.20-5.94) | <.001 | 3.53 | (3.33-3.74) | <.001 |
| No. of outpatient visits and hospitalization days | 1.01 | (1.01-1.01) | <.001 | 1.01 | (1.01-1.01) | <.001 | 1.01 | (1.01-1.01) | <.001 |
| ^a^major adverse cardiovascular event.  ^b^hazard ratio.  ^c^confidence intervals.  ^d^hypertension.  ^e^diabetes mellitus.  ^f^medication possession ratio.  ^g^Medication non-user who have never been prescribed. | | | | | | | | | |
